# Transmission of foreshock waves through Earth’s bow shock

**DOI:** 10.1038/s41567-022-01837-z

**Published:** 2022-12-19

**Authors:** L. Turc, O. W. Roberts, D. Verscharen, A. P. Dimmock, P. Kajdič, M. Palmroth, Y. Pfau-Kempf, A. Johlander, M. Dubart, E. K. J. Kilpua, J. Soucek, K. Takahashi, N. Takahashi, M. Battarbee, U. Ganse

**Affiliations:** 1 Department of Physics, University of Helsinki, Helsinki, Finland; 2 Space Research Institute, Austrian Academy of Sciences, Graz, Austria; 3 Mullard Space Science Laboratory, University College London, Dorking, UK; 4 Swedish Institute of Space Physics, Uppsala, Sweden; 5 Departamento de Ciencias Espaciales, Instituto de Geofísica, Universidad Nacional Autónoma de México, Mexico City, Mexico; 6 Finnish Meteorological Institute, Helsinki, Finland; 7 Institute of Atmospheric Physics, Czech Academy of Sciences, Prague, Czech Republic; 8 The Johns Hopkins University Applied Physics Laboratory, Laurel, MD USA; 9 Department of Earth and Planetary Science, Graduate School of Science, University of Tokyo, Tokyo, Japan; 10 Radio Research Institute, National Institute of Information and Communication Technology, Tokyo, Japan

**Keywords:** Magnetospheric physics, Magnetospheric physics

## Abstract

The Earth’s magnetosphere and its bow shock, which is formed by the interaction of the supersonic solar wind with the terrestrial magnetic field, constitute a rich natural laboratory enabling in situ investigations of universal plasma processes. Under suitable interplanetary magnetic field conditions, a foreshock with intense wave activity forms upstream of the bow shock. So-called 30 s waves, named after their typical period at Earth, are the dominant wave mode in the foreshock and play an important role in modulating the shape of the shock front and affect particle reflection at the shock. These waves are also observed inside the magnetosphere and down to the Earth’s surface, but how they are transmitted through the bow shock remains unknown. By combining state-of-the-art global numerical simulations and spacecraft observations, we demonstrate that the interaction of foreshock waves with the shock generates earthward-propagating, fast-mode waves, which reach the magnetosphere. These findings give crucial insight into the interaction of waves with collisionless shocks in general and their impact on the downstream medium.

## Abstract (web summary)

The Earth’s bow shock results from the interaction of the solar wind with the terrestrial magnetic field. With global numerical simulations and spacecraft observations, the transmission of fast magnetosonic waves through the bow shock is revealed.

## Main

Collisionless super-critical shocks are highly efficient particle accelerators observed throughout the universe. When the shock geometry is quasi-parallel, that is, when the angle *θ*_*Bn*_ between the upstream magnetic field and the shock normal is below 45°, shock-reflected particles can travel far upstream and excite instabilities, forming an extended foreshock or precursor hosting intense wave activity. These waves modulate particle reflection at the shock front^1^, can affect cosmic ray acceleration at astrophysical shocks^2,3^ and cause atmospheric particle escape at non-magnetized planets^4^. The interaction of these upstream waves with the shock, their influence on particle acceleration and their transmission into the downstream medium have received considerable attention (see, for example, refs. ^1,2,5–7^), but our understanding of these processes remains limited.

**Fig. 1:**
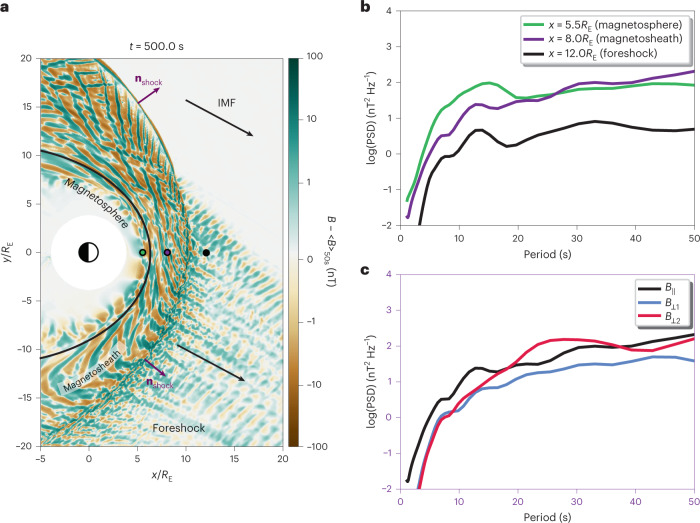
Overview of the simulation and wave activity in the foreshock and magnetosheath. **a**, Colour map of the magnetic field strength fluctuations in the simulation plane at time *t* = 500 s from the beginning of the simulation. We subtract <*B*>_50s_, which is a 50 s average of the field magnitude, from *B* to reveal the fluctuations of the magnetic field magnitude. The black curve shows the approximate magnetopause location. The black arrows show the IMF direction, and the purple arrows depict the shock normal direction **n**_shock_ at two positions along the bow shock. **b**, PSD of the total magnetic field fluctuations at the three locations marked by coloured circles in **a**. **c**, PSD of the magnetic field fluctuations parallel and perpendicular to the mean magnetic field at the virtual spacecraft location in the magnetosheath. The perpendicular directions are defined such that *B*_⊥1_ lies in the simulation (*x–y*) plane while *B*_⊥2_ completes the right-handed set.

At Earth, ultra-low-frequency waves originating in the foreshock are considered the main source of magnetospheric Pc3 waves (22−100 mHz), suggesting wave transmission^8–11^. Compressional Pc3 waves are routinely observed in the dayside magnetosphere, where they couple with field-line resonances^12^, forming a remote diagnostic of magnetospheric density through magnetoseismology^13^. They also modulate energetic particle precipitation into the upper atmosphere^14,15^. Nevertheless, after decades of intensive research, it is still unclear how foreshock waves traverse through the bow shock and the downstream magnetosheath, populated by shocked solar wind plasma (Fig. 1a).

This paper addresses the important question of the interaction of low-frequency waves with a collisionless shock and presents the missing link in foreshock wave transmission. This discovery was sparked by large-scale numerical simulations, showing the global picture of wave transmission. This allows us to identify relevant observations from the Magnetospheric Multiscale (MMS) mission^16^, which reveal the presence of fast magnetosonic waves in the subsolar magnetosheath, with properties consistent with a foreshock source. Our findings provide compelling evidence of the process connecting foreshock and magnetospheric waves.

Foreshock ‘30 s waves’, named after their typical period at Earth, are fast magnetosonic waves generated by cyclotron-resonant instabilities driven by shock-reflected particles in the solar wind^17–20^. They play an important role in modulating the shape of the shock front^5^, affecting particle reflection at the shock^1^ and contributing to quasi-parallel shock reformation^7^. Two main observations support the connection between foreshock 30 s waves and Pc3 fluctuations: (1) in both regions, the waves have very similar frequencies showing the same dependency on the interplanetary magnetic field (IMF) strength^8,9,21^, and (2) magnetospheric Pc3 wave power recedes when the IMF cone angle, measured from the Sun–Earth line, increases, causing the foreshock to shift away from the subsolar point^8,11,22^.

Direct transmission of the waves through the bow shock and magnetosheath was initially envisioned^22^ and is still to date widely invoked^23–25^. However, the lack of observational evidence for fast-mode waves of foreshock origin in the magnetosheath, despite extensive surveys (see, for example, refs. ^26,27^), has cast doubt on this scenario. Furthermore, early numerical works suggested that foreshock waves mode-convert into Alfvén/ion cyclotron waves upon crossing the bow shock^28,29^ and thus could not transmit as fast-mode waves. More indirect pathways have been explored, for example, localized variations of magnetosheath dynamic pressure that would cause magnetopause motions^30^, or modulated precipitation into the ionosphere^31^, but no consensus has been reached, and this long-standing question remains unsolved.

Here, we explore this issue using state-of-the-art global ion kinetic simulations performed with Vlasiator, a hybrid-Vlasov model designed to simulate the near-Earth plasma environment (refs. ^32,33^ and Methods). Vlasiator has the unique capability of providing a global view of near-Earth space while including ion kinetic processes with correct scale separation, allowing for direct comparison with spacecraft observations. It has been extensively used to study foreshock processes, providing excellent agreement with observational works^19,20,34–38^, and we now apply it to the study of foreshock wave transmission. We analyse the same run as presented in ref. ^38^, with upstream conditions corresponding to those encountered at Earth on 20 July 2016 between 08:00 and 12:00 universal time. At that time, near-Earth space was engulfed in a large-scale solar wind structure, a magnetic cloud^39^, causing a long-lasting interval of steady solar wind parameters. Because of the magnetic cloud, the solar wind parameters deviate from their typical values (solar wind density *n* = 12 cm^−3^, velocity **V** = (−565, 0, 0) km s^−1^ and IMF vector **B** = (12.5, −6.5, 0) nT, with a 28° cone angle). The values of the magnetosonic and Alfvén Mach numbers (*M*_ms_ = 5.5 and *M*_A_ = 6.4, respectively) are, however, typical for Earth’s bow shock^40^. We select this run because the large amplitude of the foreshock waves^38^ facilitates their tracking across near-Earth space.

Large fluctuations of the magnetic field strength are observed throughout the simulation domain (Fig. 1a). Foreshock compressive fluctuations become stronger when approaching the shock, in agreement with spacecraft observations^41^. In the magnetosphere, the magnetic field variations decay when moving inwards, consistent with the attenuation of compressive waves propagating into the magnetosphere^25,38^.

Figure 1b shows the power spectral density (PSD) of the magnetic field strength fluctuations obtained from a wavelet transform of the time series extracted at the positions marked by the coloured circles in Fig. 1a. The PSD shows a clear peak at ~13 s in the foreshock (black curve) and in the magnetosheath (purple curve). The period of the peak agrees well with the expected wave period for these solar wind conditions (12 s according to the Takahashi et al.^9^ formula), below the typical 30 s because of the high IMF strength^20^. The wave power increases by a factor of ~5 from the foreshock to the magnetosheath, in agreement with ref. ^42^. A somewhat broader peak in the same period range, from 5 to 20 s, is found in the outer magnetosphere (green curve), where the wave power is further amplified by a factor of ~4.

**Fig. 2:**
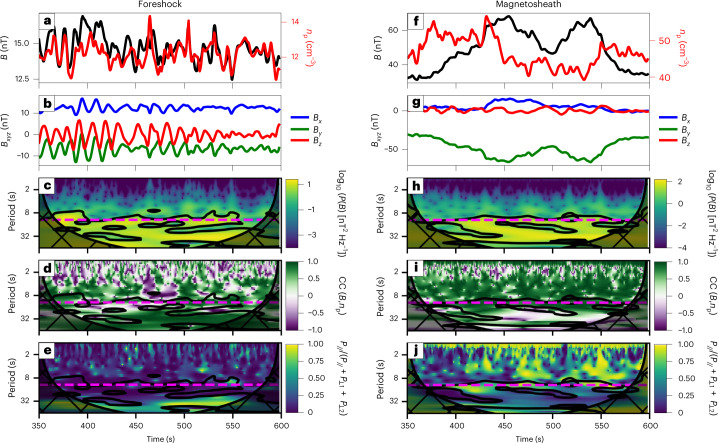
Virtual spacecraft observations in the foreshock and magnetosheath. **a**,**b**,**f**,**g**, Time series of the magnetic field strength and ion density (**a** and **f**), and of the magnetic field components (**b** and **g**). **c**–**e**,**h**–**j**, Wavelet power spectrum of the magnetic field strength where P is the wave power (**c**) and (**h**), wavelet cross-correlation (CC) of the magnetic field strength and density fluctuations (**d** and **i**), and compressibility of the magnetic field fluctuations, defined as the wave power parallel to the mean magnetic field P_//_ divided by the total wave power P_//_+ P_⊥1_+ P_⊥2_ (**e** and **j**). The data were extracted at (*x* = 12*R*_E_, *y* = 0*R*_E_) (left) and (*x* = 8*R*_E_, *y* = 0*R*_E_) (right). The dashed pink line in **c**–**e** and **h**–**j** shows the foreshock wave period predicted using the Takahashi et al.^9^ formula. Note that the time series used for the magnetosheath wavelet power spectra have been high-pass filtered to remove low-frequency variations due to boundary motion (with a cut-off at 40 s), to better highlight the wave power in the relevant period range. The hatched area in **c**–**e** and **h**–**j** shows the cone of influence, where edge effects are dominant, while the solid black line marks the 95% significance level. Source data

Figure 2 shows the corresponding time series in the foreshock and magnetosheath. There is a large wave power near the predicted foreshock wave period (Fig. 2c,h), with some variability probably due to the large IMF strength causing a more complex wave field^20^. These magnetic field strength fluctuations are associated with density variations (Fig. 2a,f). Both are mostly positively correlated near the foreshock wave period (Fig. 2d,i), indicative of fast-mode fluctuations and ruling out mirror-mode waves. In the foreshock, the fluctuations near 12 s are only weakly compressional (Fig. 2e), consistent with 30 s wave properties^43^. In contrast, the wave power near 30−40 s is associated with more compressional waves and thus a different wave mode. In the magnetosheath, the fluctuations at the foreshock wave period are strongly compressional (Figs. 2j and 1c).

We apply multi-spacecraft timing analysis^20,44^ to the magnetic field measurements from a triplet of virtual spacecraft around *x* = 8*R*_E_ (Earth radius = 6371 km) (Fig. 1a, purple dot) with a spacecraft separation of ~0.05*R*_E_, and find earthwards-oriented wavevectors lying at about 10° from the Sun–Earth line. The associated *θ*_*kB*_ angle, measured between the wavevector and the ambient magnetic field, is close to 90°. The wave velocity is within 20% of the local fast magnetosonic speed.

**Fig. 3:**
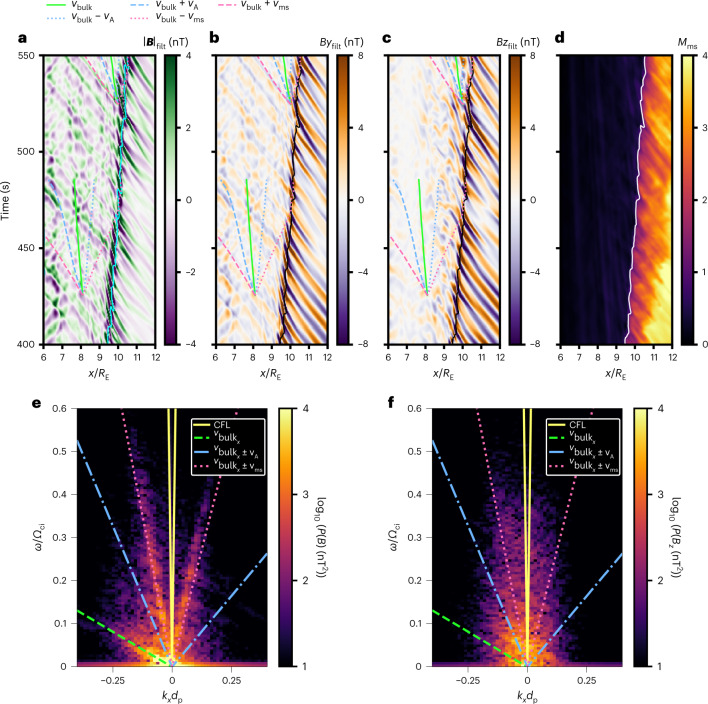
Wave activity along the Sun–Earth line. **a**–**c**, Maps of the magnetic field strength (**a**) and its *B*_*y*_ (**b**) and *B*_*z*_ (**c**) components along the Sun–Earth line, as a function of *x* and time. The data have been high-pass filtered, with a cut-off at 40 s, to highlight the relevant frequencies, as indicated by the subscript “filt” in the variable names. The negative magnetic field strength values are due to this filtering. The cyan contour in **a** marks where the ion density reaches twice its solar wind value, a proxy for the shock position. The black contour in **b** and **c** marks where the magnetosonic Mach number *M*_ms_ = 1. The coloured lines in the magnetosheath indicate streamlines originating from two locations associated with typical plasma velocities: bulk speed (green), Alfvén speed (blue) and fast magnetosonic speed (pink). The dashed lines correspond to an earthwards propagation in the plasma rest frame, and the dotted lines to a sunwards propagation. The outward motion of the bow shock is due to the two-dimensional (2D) setup of our simulation, as interplanetary magnetic field lines pile up in front of the magnetosphere. **d**, Time–position map of the magnetosonic Mach number. The white contour marks where *M*_ms_ = 1. **e**,**f**, Dispersion plots obtained from the 2D Fourier transform of the magnetic field strength (**e**) and *B*_*z*_ component (**f**) between *x* = 6.5*R*_E_ and *x* = 9*R*_E_, using unfiltered data to which a Hann window has been applied along both dimensions. On the horizontal axis, the frequencies are normalized to the ion cyclotron frequency *Ω*_ci_ and the wavenumber, on the vertical axis, to the proton inertial length *d*_p_. The solid yellow lines show the Courant–Friedrichs–Lewy (CFL) condition, which is the maximum speed at which information can travel in the simulation. The median bulk speed in the magnetosheath, at the locations used to calculate the 2D Fourier transform (${v}_{{{{{\rm{bulk}}}}}_{x}}=48$ km s^−1^) is indicated by the dashed green line. The dash-dotted blue lines and the dotted pink lines indicate sunwards and earthwards propagation at the median Alfvén speed (*v*_A_ = 145 km s^−1^) and median fast magnetosonic speed (*v*_ms_ = 360 km s^−1^), respectively, in the plasma rest frame.

Figure 3a shows a time–position map of the magnetic field strength along the Sun–Earth line. The foreshock waves appear as alternating bands of purple and green in the right-hand part of the plot, moving earthwards as time progresses. Similar features are seen for the *B*_*y*_ and *B*_*z*_ components (Fig. 3b,c, blue and orange bands). Figure 3a,b shows waves in the magnetosheath with approximately the same slope as their upstream counterparts, extending to the magnetopause, located around *x* = 6*R*_E_. In contrast, the *B*_*z*_ fluctuations lose their coherency, suggesting a different wave polarization. In the magnetosheath, the waves are polarized along the magnetic field (*B*_*y*_ being the dominant component), different from their circular polarization in the foreshock (shown, for example, in refs. ^17,19^).

These waves propagate earthwards at the fast magnetosonic speed in the magnetosheath plasma rest frame, as indicated by their slope in the time–position map following the dashed pink lines, corresponding to the sum of the fast magnetosonic and plasma bulk speed. In addition to these fast-mode waves, Fig. 3a–c also shows structures advected by the magnetosheath flow (almost vertical lines, following the green lines), and disturbances propagating from the magnetopause to the bow shock at the fast magnetosonic speed (coloured stripes with a positive slope), which may be due to the reflection of incoming waves at the magnetopause.

**Extended Data Fig. 1:**
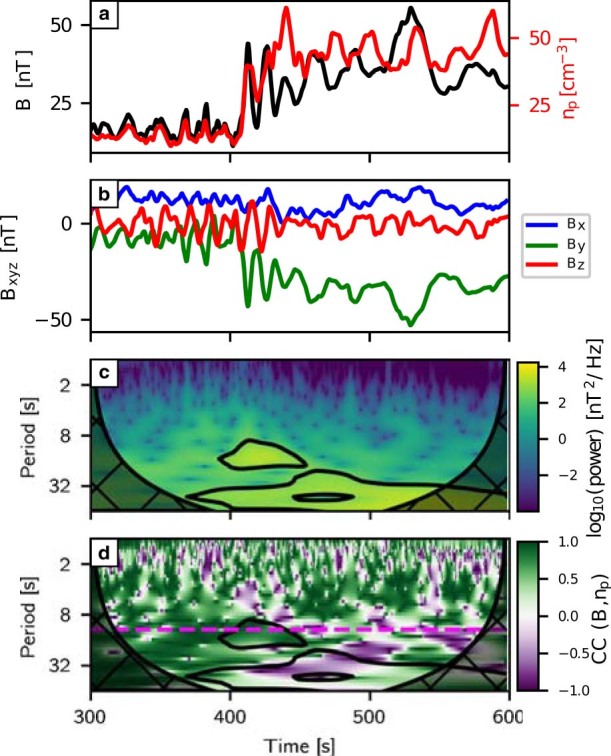
Virtual spacecraft observations near the bow shock. From top to bottom: (a) magnetic field strength (black) and ion density (red), (b) magnetic field components, (c) wavelet power spectrum of the magnetic field strength, and (d) cross-correlation of the magnetic field strength and density fluctuations. The pink dashed line in panel d shows the predicted foreshock wave period using the Takahashi et al.^9^ formula. The black contours in panels c and d delineate the 95% confidence interval of the wavelet power spectrum. The virtual spacecraft is positioned in (*x* = 9.6 R_E_; *y* = 0 R_E_) and is initially located in the foreshock, then crosses the shock shortly after *t* = 400 s and remains in the magnetosheath afterwards, due to the outward bow shock motion. Between *t* = 400 − 450 s, fast-mode oscillations at the foreshock wave period are observed just downstream of the shock, consistent with the observations reported by Liu et al.^7^. Source data

As they approach the bow shock, foreshock waves participate in shock reformation, consistent with spacecraft observations^7^ (see also Extended Data Fig. 1). This is illustrated in Fig. 3a by the quasi-periodic motion of the density contour which serves as a proxy of the shock position^45^. The fast-mode structures identified by ref. ^7^ in the immediate downstream are the nearly vertical stripes within ~0.3*R*_E_ from the shock in Fig. 3a–c (see Extended Data Fig. 1 for virtual spacecraft time series allowing direct comparison with MMS observations in ref. ^7^). Their propagation speed is close to the bulk flow speed, in good agreement with ref. ^7^. Although these structures are prominent in the immediate shock vicinity, they quickly dissipate, while those disturbances travelling earthwards at the fast magnetosonic speed become more visible further downstream.

**Extended Data Fig. 2:**
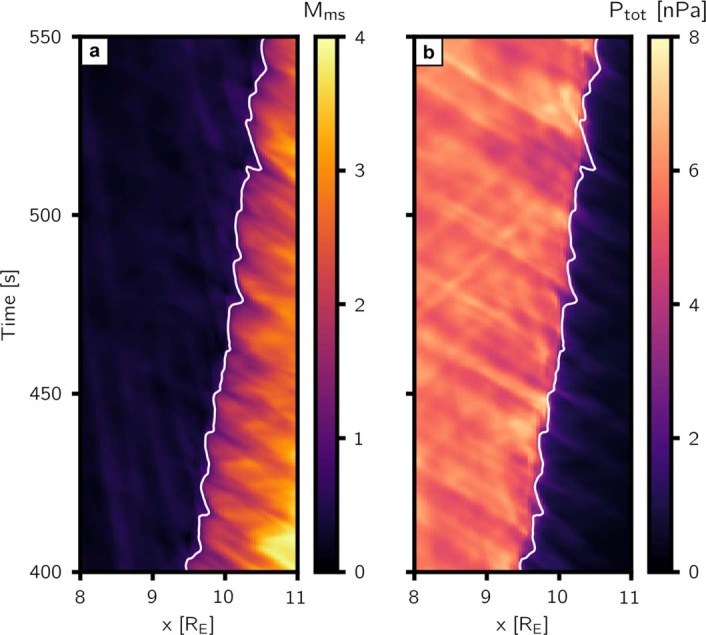
Total pressure variations in the magnetosheath caused by the foreshock waves. Time-position maps of the magnetosonic Mach number (a) and the total pressure (b) along the Sun-Earth line. The total pressure is calculated as the sum of the thermal pressure and the magnetic pressure. The white contour marks where the magnetosonic Mach number Mms = 1.

The foreshock waves further modulate the magnetosonic Mach number upstream of the shock (Fig. 3d) and consequently the shock compression ratio. This results in total pressure variations in the downstream (Extended Data Fig. 2), which are probably the source of the fast-mode waves traversing the magnetosheath.

To further confirm the nature of these waves, Fig. 3e shows the wave power in (*ω*, *k*_*x*_) space obtained from the two-dimensional (2D) Fourier transform of Fig. 3a between *x* = 6.5*R*_E_ and *x* = 9*R*_E_. We find a large wave power in fluctuations propagating earthwards (that is, with negative *k*_*x*_) with the fast magnetosonic speed. This lends further support to the foreshock waves traversing the magnetosheath as fast-mode waves. Again, we note that these waves are accompanied by structures travelling at the plasma bulk speed (along the dashed green line) and sunwards-propagating fluctuations at the fast magnetosonic speed (along the pink line at positive *k*_*x*_). The transverse wave power (Fig. 3f) suggests that there could be upstream-propagating Alfvén waves, confined to low frequencies (Extended discussion of Vlasiator 1D simulation section).

**Extended Data Fig. 3:**
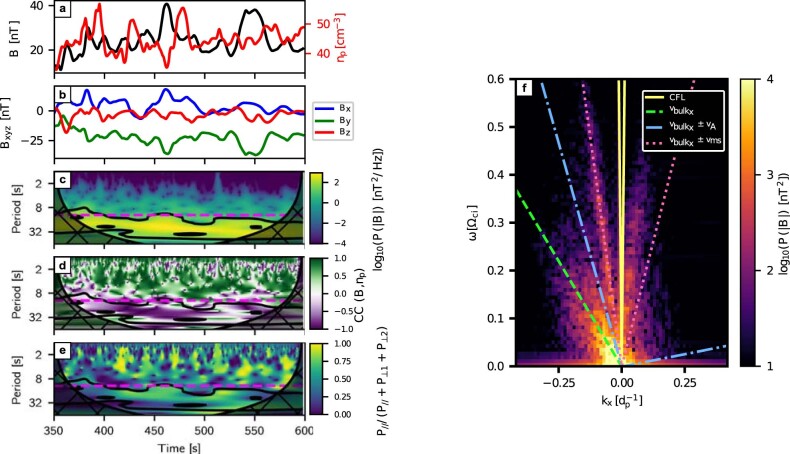
Magnetosheath wave activity away from the Sun-Earth line. Left-hand side: time series of the magnetic field and plasma parameters extracted at a virtual spacecraft positioned in (*x* = 8 R_E_; *y* = -5 R_E_). The panels show, from top to bottom: (a) the magnetic field strength (in black) and the ion density (red), (b) the magnetic field components, (c) the wavelet power spectrum of the magnetic field strength, (d) the cross-correlation of the magnetic field strength and density fluctuations, and (e) the compressibility of the magnetic field fluctuations, defined as the wave power parallel to the mean magnetic field divided by the total wave power. The pink dashed line in panel d shows the predicted foreshock wave period using the Takahashi et al.^9^ formula. The black contours in panels c-e delineate the 95% confidence interval of the wavelet power spectrum and the hatched area marks the cone of influence. The time series used for panels c-e have been high-pass filtered to remove low frequency variations due to boundary motion (with a cutoff at 40 s), to better highlight the wave power in the relevant period range. Right-hand side: Dispersion plot obtained from the 2D Fourier transform of the magnetic field strength in the magnetosheath between *x* = 5 R_E_ and *x* = 8 R_E_ at *y* = - 5 R_E_ using unfiltered data to which a Hann window has been applied along both dimensions. On the horizontal axis, the frequencies are normalised to the ion cyclotron frequency Ω_ci_ and the wave number, on the vertical axis, to the proton inertial length d_p_. The solid yellow lines show the Courant-Friedrichs-Lewy condition, which is the maximum speed at which information can travel in the simulation. The median bulk speed in the magnetosheath, at the locations used to calculate the 2D Fourier transform is indicated by the green dashed line. The blue dash-dotted lines and the pink dotted lines indicate sunward and earthward propagation at the median Alfvén speed and median fast-magnetosonic speed, respectively, in the plasma rest frame.

Similar dispersion plots are obtained when calculating the 2D Fourier transform along other cuts crossing the quasi-parallel magnetosheath further down on the flank (see the right-hand side of Extended Data Fig. 3 for an example), revealing that the wave transmission is not limited to the Sun–Earth line. However, the PSD peak associated with the magnetosheath fast-mode waves in virtual spacecraft data tends to disappear when moving away from the subsolar region, suggesting that the waves would not be identified in spacecraft measurements. This is likely due to other magnetosheath waves with comparable or higher power dominating the power spectrum and concealing the magnetosheath fast-mode waves.

Based on our simulation results, we analyse observations from the MMS mission^16^ in the subsolar region, downstream of the quasi-parallel shock, to test our numerical predictions. From 2015 to 2020, we identified intervals with compressional magnetic field fluctuations at periods consistent with those of foreshock waves. The determination of the wave properties (Methods) further required that high time resolution burst mode data were available, reducing our data set to seven intervals. For each interval, the period of the foreshock waves is obtained from a theoretical formula^9^. Three of the seven comprise direct foreshock wave observations shortly before or after the magnetosheath intervals, confirming the validity of the predicted wave period. The times of the events and the associated IMF conditions are listed in Extended Data Table 1. All intervals but one are associated with an IMF cone angle around 30−40° (which, in the subsolar region, approximates well the *θ*_*Bn*_ angle at the shock upstream of the spacecraft) and are thus in a geometry similar to that in our numerical analysis.

**Fig. 4:**
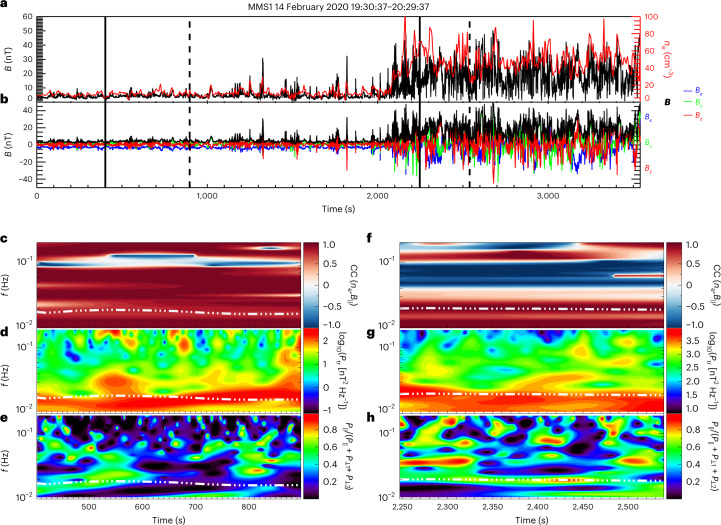
MMS observations in the foreshock and the magnetosheath on 14 February 2020. **a**,**b**, The magnetic field strength (black) and electron density (red) (**a**) and the magnetic field components (**b**) as functions of time. **c**–**h**, Wave properties during two sub-intervals marked with vertical solid and dashed lines in **a** and **b** in the foreshock (**c**–**e**) and the magnetosheath (**f**–**h**): the cross-correlation between the magnetic field strength and electron density fluctuations (**c** and **f**), the wavelet trace power (*P*_tr_) spectrum (**d** and **g**) and the magnetic field compressibility, defined as the power of the magnetic field fluctuations along the mean magnetic field direction *P*_∥_ divided by the total magnetic field wave power (**e** and **h**). The dot-dashed lines denote the expected foreshock wave frequency.

**Extended Data Fig. 4:**
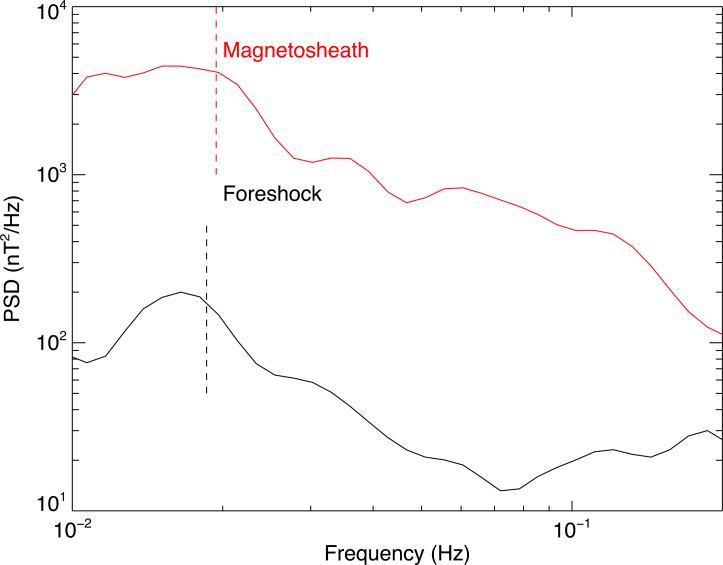
Power spectral density of the foreshock and magnetosheath fluctuations observed by MMS on 14 February 2020. Power spectral density of the total magnetic field fluctuations calculated for the intervals marked by the vertical lines in Fig. 4 in the foreshock (black) and in the magnetosheath (red). The vertical dashed lines indicate the predicted foreshock wave frequency for each interval from the Takahashi et al.^9^ formula.

Figure 4 shows MMS1 observations during one representative interval, when the spacecraft moved from the foreshock into the subsolar magnetosheath, downstream of the quasi-parallel shock, on the inbound leg of its orbit on 14 February 2020. In the foreshock, MMS1 observed weakly compressional waves near the expected foreshock wave frequency, with properties consistent with fast-mode 30 s waves (Fig. 4c–e). Shortly thereafter, waves at similar frequencies were encountered in the magnetosheath (see Extended Data Fig. 4 for the PSD), again associated with positively correlated density and magnetic field fluctuations and this time with a stronger compressive component (Fig. 4f–h), consistent with the properties of the fast-mode waves in our numerical analysis.

**Fig. 5:**
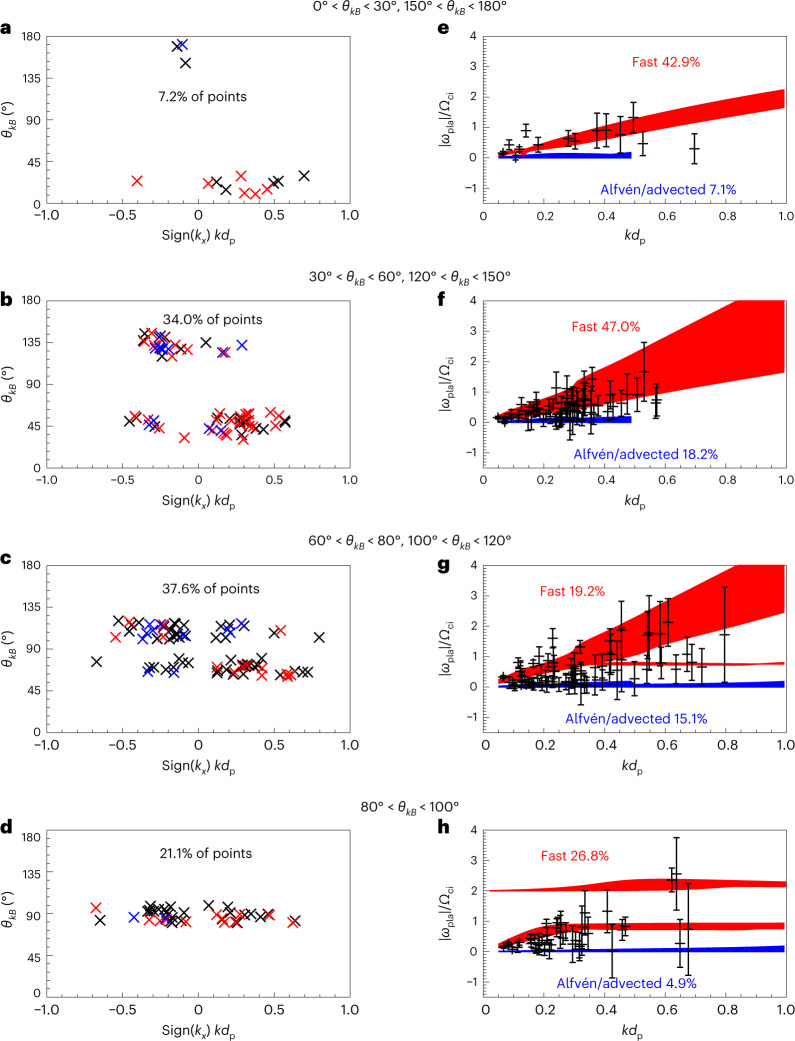
Experimental wave properties obtained from MMS observations. The data set includes all events listed in Extended Data Table 1. **a**–**d** Orientation of the wavevectors, with negative *k*_*x*_ corresponding to earthwards propagation, as a function of the angle *θ*_*kB*_ between the wavevector and magnetic field. The data are divided between four ranges of *θ*_*kB*_ values to distinguish between nearly parallel (*θ*_*kB*_ ≈ 0° or *θ*_*kB*_ ≈ 180°), nearly perpendicular (*θ*_*kB*_ ≈ 90°) and intermediate propagation direction. The percentage in each panel indicates the fraction of data points within this *θ*_*kB*_ range. The data points marked in red (blue) correspond to those points found within the red (blue) areas in **e**–**h** and are thus consistent with the fast wave (Alfvén wave) solution from linear Vlasov theory. The points outside both areas are left in black. **e**–**h**, Recovered plasma frame wave frequencies (normalized to the ion cyclotron frequency *Ω*_ci_) as a function of the wavevectors (normalized to the proton inertial length *d*_p_). These are separated by the orientation of the wavevector with respect to the mean-field direction. The red areas denote the fast wave solutions from linear Vlasov theory, and the blue areas denote the solutions expected for the Alfvén wave solutions. The percentages in red (blue) indicate the fraction of data points found within the red (blue) area. The solutions are calculated using the extreme *θ*_*kB*_ values for each angle range and isotropic ion and electron temperatures. The extremes of proton and electron plasma *β* are *β*_p_ = [5, 20], and *β*_e_ = [1, 3], and the ratio of Alfvén speed to the speed of light is 2 × 10^−4^. The error bars on *ω* are derived from the s.d. of the velocity component in the direction of the obtained wavevector.

For this magnetosheath interval and for all other selected intervals (Extended Data Table 1), we calculate the wavevector and frequency in the plasma rest frame using a single-spacecraft method based on magnetic field and current density measurements (ref. ^46^ and Methods). We restrict our analysis to spacecraft frame frequencies below 0.1 Hz, that is, to the frequency range of the waves of interest. The results of this analysis for the seven magnetosheath intervals are displayed in Fig. 5. Figure 5a–d shows that the waves propagate at oblique to large angles with respect to the ambient magnetic field (*θ*_*kB*_ > 30°), and that there are both earthwards- (negative *k*_*x*_) and sunwards-oriented (positive *k*_*x*_) wavevectors in the plasma rest frame.

**Extended Data Fig. 5:**
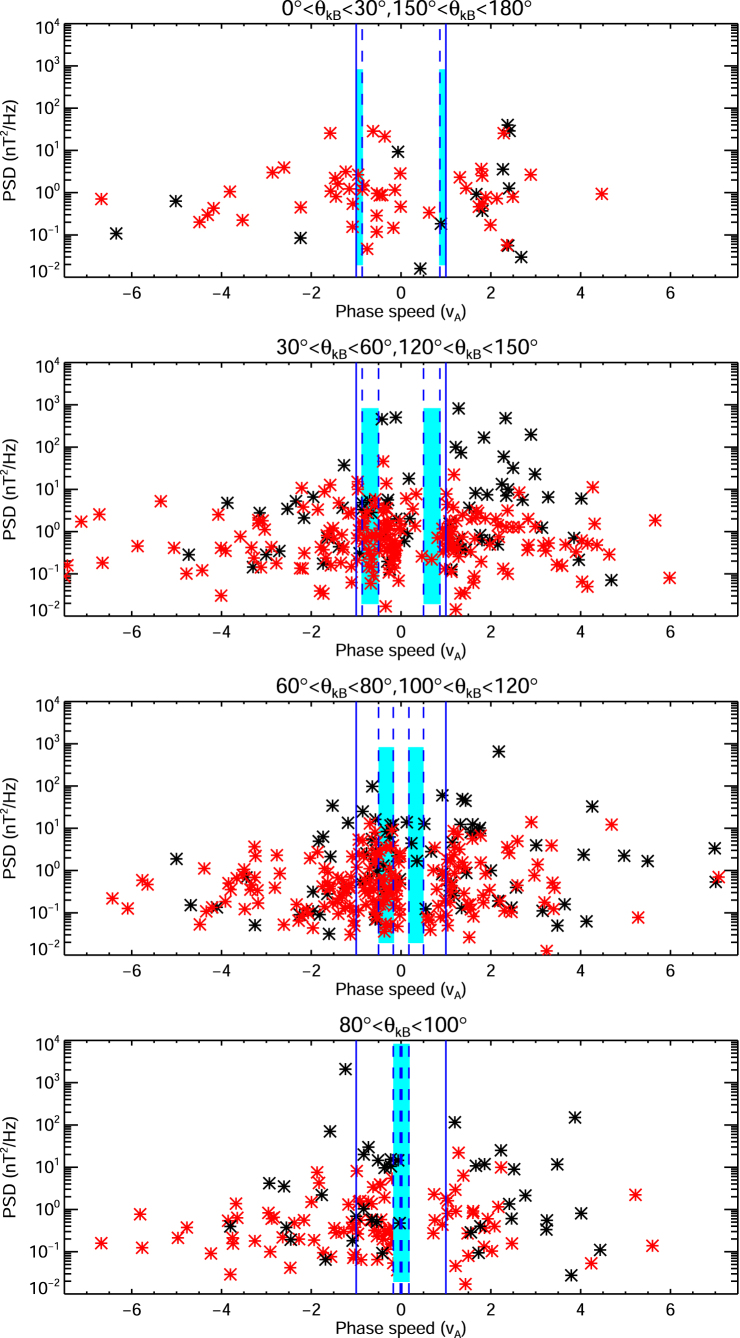
Wave phase speeds from MMS observations. Total magnetic power as a function of the wave phase speeds *v*_ph_ = *ω*/k. The phase speeds correspond to the recovered points obtained from the Bellan method and are organised as a function of the orientation of the wavevector, with a negative speed indicating earthward propagation. The data points in black are the same as shown in Fig. 5, for which the current density was obtained from the particle measurements. Those in red are based on the current density calculated using the curlometer method. The results from both approaches are in excellent agreement. The vertical blue line denotes the Alfvén speed. The dashed lines denote the phase speeds for largest and smallest phase speeds ${v}_{{{{\rm{A}}}}}\cos ({\theta }_{{{{\rm{kB}}}}})$ and the shaded cyan area denotes the region where the speeds are consistent with Alfvén waves or advected structures.

Figure 5e–h shows the experimental (*ω*, *k*) values in the plasma frame, separated into different ranges of *θ*_*kB*_. We compare them with the linear solutions for the fast (red) and Alfvén (blue) modes obtained from linear Vlasov theory^47^. The percentages in Fig. 5e–h indicate which fraction of the data points are within the red and blue areas, which account for the different plasma conditions in the intervals. We find that a large fraction of the data points show good agreement with the fast-mode solution, while only fewer data points are closer to the Alfvén solution. This suggests that both modes co-exist in the magnetosheath, with the fast mode being predominant. Those data points that fall within the red/blue areas in Fig. 5e−h are marked with the same colours in Fig. 5a–d, suggesting that there are both earthwards- and sunwards-propagating fast-mode waves in the magnetosheath, as reported in our simulation (Fig. 3). Furthermore, the Alfvén solutions exhibit low frequency and, considering the error bars, may in fact be advected structures with no intrinsic frequency. Finally, the wave phase speed (Extended Data Fig. 5) is generally larger than the Alfvén velocity, bringing further support to the presence of fast-mode waves in the magnetosheath during these intervals.

In summary, MMS observations show the presence of earthwards-propagating fast-mode waves in the quasi-parallel subsolar magnetosheath, at frequencies matching those of foreshock waves, in agreement with our model predictions. Our numerical and observational results, therefore, provide strong evidence that foreshock waves traverse the magnetosheath as fast magnetosonic waves, as was first inferred to explain the occurrence of magnetospheric Pc3 waves (see, for example, ref. ^22^). Our findings regarding the wave frequency and their compressional nature in the magnetosheath are in excellent agreement with their entry into the magnetosphere as fast-mode Pc3 waves^15,23,25^.

**Fig. 6:**
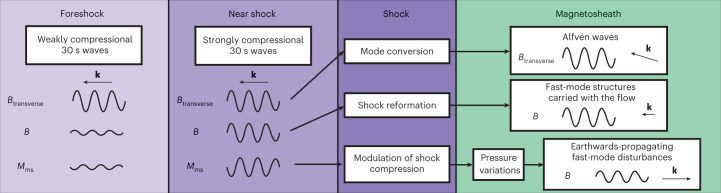
Schematic of the interaction of foreshock waves with the shock. Summary of our findings and the scenario we propose for the interaction of foreshock waves with the shock and the resulting waves and structures in the magnetosheath. The wave propagation is shown from left to right, from the foreshock away from the shock (light purple) to the magnetosheath (green). The relevant properties of the 30 s waves are indicated in the foreshock (left) and just upstream of the shock (second from the left). The processes occurring upon their interaction with the shock are marked in the third box (dark purple). The resulting waves and structures in the magnetosheath are listed in the rightmost box (green).

However, despite these similarities with previous works, we also find that the wave propagation through the bow shock is more complex than the typically inferred direct transmission. The earthwards-oriented wavevector of the magnetosheath fast-mode waves is not consistent with that of directly transmitted foreshock waves, indicating that new waves are generated at the shock by a process modulated by the foreshock waves. These downstream waves transmit the information of the wave period through the magnetosheath, thus providing the missing link between foreshock and magnetosphere. We propose the following scenario for the downstream wave generation (see also Fig. 6): Foreshock waves modulate the magnetosonic Mach number upstream of the shock and consequently the shock compression ratio. An increased (decreased) compression ratio creates a zone of enhanced (reduced) pressure just downstream of the shock. It is then this pressure imbalance that generates fast-mode compressive/rarefaction waves travelling through the magnetosheath, in a process similar to that described by Wu et al.^48^ for discontinuities interacting with a shock in magnetohydrodynamic simulations, confirmed observationally^49^. The discontinuities in the Wu et al.^48^ study are comparable with the foreshock fast-mode waves in that they also cause a change in the upstream magnetosonic Mach number.

The hybrid simulations by Thomas et al.^50^ show that downstream-oriented fast-mode waves are also generated when pressure pulses, propagating upstream in the plasma rest frame as in the present work, hit the shock. These earthwards-propagating fast-mode waves are accompanied by mode-converted Alfvén waves in the downstream, indicating that both wave modes co-exist in their simulations as well^50^. As noted by the authors, the pressure pulses in their simulation correspond to another type of foreshock waves^50^. Our work expands upon their study in demonstrating that the interaction of 30 s waves with the shock produces similar downstream waves, and in unravelling the processes taking place at the shock. The generation of fast-mode waves in the downstream requires that foreshock waves are compressional just upstream of the bow shock, which is typically the case at Earth (see, for example, ref. ^41^). Although the scenario we propose is based on a fluid description, the downstream fast-mode waves were not predicted by previous theoretical works (see, for example, ref. ^51^), possibly because of their indirect generation, in the downstream of the shock, or because of the linear approximation used in these works, which implies that the waves are only weakly compressional.

**Extended Data Fig. 6:**
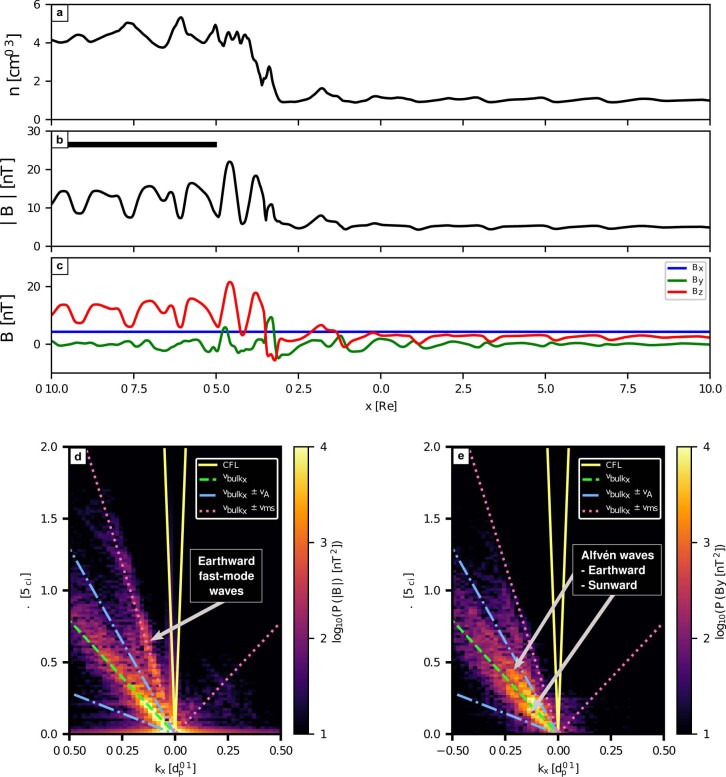
Wave activity in a 1D local shock simulation with Alfvén Mach number M_A_ = 6.9. Proton density (a), magnetic field strength (b) and magnetic field components (c) along the *x* axis at t = 500 s from the beginning of the simulation. (d) and (e) Dispersion plots obtained from the 2D Fourier transform of the magnetic field strength (d) and By component (e) between *x* = -10 R_E_ and *x* = -5 R_E_ (marked by the black bar in panel (b)) and t = 250 - 500 s, using unfiltered data to which a Hann window has been applied along both dimensions. On the horizontal axis, the frequencies are normalised to the ion cyclotron frequency Ω_ci_ and the wave number, on the vertical axis, to the proton inertial length d_p_. The solid yellow lines show the Courant-Friedrichs-Lewy condition, which is the maximum speed at which information can travel in the simulation, the blue dash-dotted lines the median Alfvén speed, and the pink line the median fast-magnetosonic speed in the magnetosheath at the locations used to calculate the 2D Fourier transform. The median bulk speed is indicated by the green dashed line.

**Extended Data Fig. 7:**
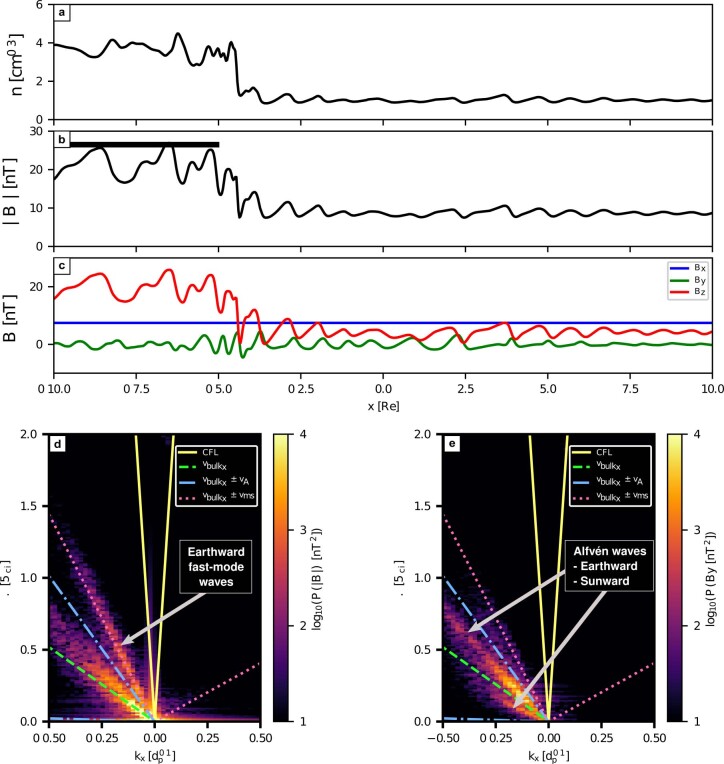
Wave activity in a 1D local shock simulation with Alfvén Mach number M_A_ = 4. Proton density (a), magnetic field strength (b) and magnetic field components (c) along the *x* axis at t = 500 s from the beginning of the simulation. (d) and (e) Dispersion plots obtained from the 2D Fourier transform of the magnetic field strength (d) and By component (e) between *x* = -10 R_E_ and *x* = -5 R_E_ (marked by the black bar in panel (b)) and t = 250 - 500 s, using unfiltered data to which a Hann window has been applied along both dimensions. On the horizontal axis, the frequencies are normalised to the ion cyclotron frequency Ω_ci_ and the wave number, on the vertical axis, to the proton inertial length d_p_. The solid yellow lines show the Courant-Friedrichs-Lewy condition, which is the maximum speed at which information can travel in the simulation, the blue dash-dotted lines the median Alfvén speed, and the pink line the median fast-magnetosonic speed in the magnetosheath at the locations used to calculate the 2D Fourier transform. The median bulk speed is indicated by the green dashed line.

To facilitate comparison with earlier works, we further performed one-dimensional (1D) shock simulations with different upstream conditions (Extended discussion of Vlasiator 1D simulation section and Extended Data Figs. 6 and 7). These simulations clearly show the co-existence of both the earthwards-propagating fast-mode disturbances reported here and the mode-converted Alfvén waves from earlier studies^28,29,52^. We also note that the fast-mode waves are not detectable in the magnetic field component transverse to both the upstream field and flow. This probably explains why the downstream fast-mode waves were not identified in previous works that focused solely on this component^28,29,52^.

Our numerical simulations show that these waves are more easily detected with the model’s global view (Fig. 3) than in time series mimicking actual spacecraft measurements. The downstream fast-mode waves appear as one of the dominant wave modes only in the subsolar magnetosheath, while they are masked by other modes further on the flanks (Extended Data Fig. 3, left). Large statistical surveys have been conducted using measurements away from the subsolar point, and often focused on the dominant wave mode^27,53,54^. This probably explains why these waves have remained elusive for so long.

The change of polarization of the fast-mode waves, from right-handed in the foreshock to linear in the magnetosheath, is due to the downstream waves being generated at the shock rather than being directly transmitted. Our global simulation demonstrates that the interaction of foreshock waves with the shock generates an array of waves in the downstream: reformation-related structures travelling with the flow (as in ref. ^7^), mode-converted Alfvén waves (as in earlier simulations^28,29,52^) and earthwards-propagating fast-mode waves (see Fig. 6 for a summary). Only the latter are responsible for the connection between the foreshock and the magnetosphere, and the generation of Pc3 magnetospheric waves.

Our results apply to super-critical collisionless shocks in general, showcasing the complexity of shock–upstream waves interactions. The consequences of our findings extend beyond near-Earth space physics, as collisionless shocks and foreshocks are ubiquitous in Solar System and astrophysical plasmas, in improving our understanding of shock processes that can affect particle acceleration^2,3^.

## Methods

### Vlasiator simulation

The numerical part of this work employs the hybrid-Vlasov model Vlasiator, targeted at global simulations of the interaction of the solar wind with the Earth’s magnetosphere^32,33^. In the hybrid-Vlasov formalism, ions are treated as velocity distribution functions, whose evolution is dictated by Vlasov’s equation, while electrons are considered as a massless charge-neutralizing fluid. Vlasov’s equation is coupled with Maxwell’s equations and Ohm’s law, with the Hall term. Vlasiator provides a self-consistent description of ion kinetic processes, such as the ion beam instabilities generating the foreshock waves of interest to the present study, in their global context.

The run presented here is performed in a 2D–3V space, that is, two dimensional in real space and three dimensional in velocity space. Vlasiator runs are computationally demanding, requiring millions of central processing unit hours and generating tens of terabytes of data even in a 2D setup. The simulation domain covers the equatorial plane of near-Earth space (*x*−*y* plane in the Geocentric Solar Ecliptic frame), extending from about −8*R*_E_ to 76*R*_E_ along *x* and from −60*R*_E_ to 31*R*_E_ along *y*. The simulation was run for 598 s. The solar wind is injected into the simulation from the +*x* boundary and can escape through the other edges of the simulation domain, at which Neumann conditions are applied. The circular inner boundary surrounding the Earth is located at 25,000 km from the Earth’s centre and is assumed to be a perfect conductor. The resolution of the simulation domain is 30 km s^−1^ in velocity space and 260 km in real space, the latter corresponding to about four times the ion inertial length in the solar wind. Previous works have shown and discussed in more detail that ion kinetic effects arise in Vlasiator even when not resolving the ion inertial length and lead to realistic foreshock dynamics^19,55^.

The solar wind is injected at the +*x* boundary as a Maxwellian distribution with density *n* = 12 cm^−3^, bulk velocity **V** = (−565, 0, 0) km s^−1^ and temperature *T* = 0.5 MK. The IMF vector **B** = (12.5, −6.5, 0) nT makes a 28° angle with the Sun–Earth line. The corresponding magnetosonic and Alfvén Mach number are *M*_ms_ = 5.5 and *M*_A_ = 6.4, respectively. All vector quantities are given in Geocentric Solar Ecliptic coordinates.

The Earth’s magnetic dipole is implemented with a realistic magnetic moment of 8.0 × 10^22^ A m^2^ and no tilt. The Earth’s dipolar field is therefore out of plane in this equatorial 2D run. Outside of the magnetosphere, in the magnetosheath and upstream of the shock, the magnetic field is dominated by the in-plane IMF. Out-of-plane components are self-consistently generated by the interaction of the IMF with the shock and the magnetosphere and by plasma instabilities.

### Wavelet analysis

To determine the properties of the magnetic field and density fluctuations in both our numerical simulations and observations, we apply a Morlet wavelet transform on the time series^56^. Wavelet analysis allows the distribution of power in time and frequency space, revealing the temporal evolution of wave activity. The magnetic compressibility (Figs. 2 and 4) is calculated as the power of the magnetic fluctuations parallel to the mean magnetic field divided by the total power of the magnetic field fluctuations. The wavelet cross-correlation computes the common power between two time series, here the electron density and the magnetic field strength, in time–frequency space (see, for example, ref. ^57^).

### Wave dispersion relation from MMS observations

To determine the wavevector from single-spacecraft observations, Bellan^46^ developed a method that uses the measured magnetic field and the plasma current derived from the density and the ion and electron velocity measurements. The wavevector, as a function of the wave frequency *ω*, is then given by

1 $${{{\bf{k}}}}(\omega )={\mathrm{i}}{\mu }_{0}\frac{{{{\bf{J}}}}(\omega )\times {{{{\bf{B}}}}}^{* }(\omega )}{{{{\bf{B}}}}(\omega )\cdot {{{{\bf{B}}}}}^{* }(\omega )},$$

where **J**(*ω*) and **B**(*ω*) refer to the Fourier transforms of the current density and the magnetic field at a spacecraft frame frequency *ω*. This method assumes quasi-neutrality and that each spacecraft frame frequency maps to a single wavevector. Using the *k*-filtering method, which can resolve multiple wavevectors at a given spacecraft frame frequency, Gershman et al.^58^ demonstrated that this approach was justified and that both methods agreed well. The unique payload on the MMS spacecraft^16^ allows the simultaneous measurement of the magnetic field from the fluxgate magnetometers^59^ and the plasma current density **J**. The high time resolution capabilities from the Fast Plasma Investigation^60^ allow the plasma current density to be measured directly as

2 $${{{\bf{J}}}}={n}_{\mathrm{e}}q({{{{\bf{v}}}}}_{{{{\bf{i}}}}}-{{{{\bf{v}}}}}_{{{{\bf{e}}}}}).$$

where *n*_e_ is the electron density, *q* the elementary charge, and **v**_**i**_ and **v**_**e**_ the ion and electron bulk velocities, respectively. Here, we focus on intervals when the MMS spacecraft are operating in burst mode. The magnetic field, electron and ion distributions are sampled at 128, 33 and 6.7 Hz, respectively. Burst mode data are needed to have ion and electron measurements at a cadence comparable to that of the magnetic field measurements. Although the waves of interest have low frequencies, high-cadence measurements allow us to better reconstruct the wave dispersion relation, in providing a broad range of frequencies. Before using equation (1), all measured quantities are resampled onto the electron plasma time tags. We use Bellan’s method as the baseline sizes of MMS are too small when compared with the waves studied. Bellan’s method is applied to MMS1–3 as some of the heads of the electron spectrometer on MMS4 have failed. The wavevectors from the three MMS observatories are averaged, and we retain only those where the differences between the three individual wavevectors at a given Fourier mode are less than 35°. Using the obtained wavevectors **k**, the ion bulk velocity **v**_i_ and the spacecraft frame frequency *ω*, the fluctuations are Doppler shifted to the plasma frame thus

3 $${\omega }_{{{{\rm{pla}}}}}=\omega -{{{\bf{k}}}}\cdot {{{{\bf{v}}}}}_{i}.$$

Some Doppler shifts result in negative frequencies. This can be interpreted by considering the phase velocity **v**_ph_ = *ω*_pla_**k**/*k*^2^ (see, for example, ref. ^61^). A negative frequency results in the direction of propagation of the wave reversing. To correct this, we reverse the sign of *ω*_pla_ and **k**.

After Doppler shifting, we plot the relation of *ω*_pla_ versus *k* and compare it with linear solutions of the Maxwell–Vlasov set of equations obtained from the New Hampshire Dispersion Relation Solver (NHDS)^47^. The NHDS solves the full hot-plasma dispersion relation for a plasma consisting of bi-Maxwellian background species for ions and electrons. For the NHDS solutions, we use the averaged observed plasma parameters as input. We calculate four dispersion relations for each branch in the given angle range, which correspond to the extremes of the ion and electron plasma *β* (that is, the dimensionless ratio of thermal to magnetic pressure) in our data set. The shaded areas in Fig. 5e–h are drawn between the highest and lowest values of these four solutions. To reduce the effects of magnetic nulls (which cause extremely large values of *β*), we calculate the median of *β* for each interval and use the limits *β*_p_ = [5, 20], and *β*_e_ = [1, 3], and isotropic ion and electron temperatures to calculate the dispersion relations.

For parallel propagation, the Alfvén branch describes the ion cyclotron wave and is heavily damped under these plasma conditions. The parallel fast-mode branch is not as heavily damped and can exist to larger wavenumbers. For quasi-perpendicular propagation, the Alfvén branch describes the kinetic Alfvén wave, which has a very low phase speed and can exist to larger wavenumbers. The fast branch transitions to the ion Bernstein wave at harmonics of the ion cyclotron frequency.

As mentioned above, the Bellan^46^ method assumes that there is only one **k** vector associated to each Fourier mode, which is probably not the case here (Fig. 3d). As a result, this method may alternately pick up wavevectors from the different co-existing modes at the same Fourier mode. The *k*-filtering method does not have this limitation, but cannot be used here because the spacecraft separation is too small compared with the wavelength, resulting in large errors in the determination of the wave properties^62^.

We compared the currents obtained from the plasma measurements with those from the curlometer method^63^ and found them to be in good agreement. We also calculated the wave properties using the Bellan method applied to the current estimates from the curlometer, which confirmed the presence of earthwards-propagating fast-mode waves in the magnetosheath. The wave phase speeds derived from the curlometer currents are shown in Extended Data Fig. 5.

### Extended discussion of Vlasiator 1D simulation

In addition to the global simulation presented in the manuscript, we also carried out local 1D shock simulations to investigate the wave transmission in a set-up more similar to that used in previous numerical works^28,29,52^. The single spatial dimension is along the shock normal, while the velocity space remains three dimensional. The upstream conditions are set to *n* = 1 cm^−3^, **V** = (−750, 0, 0) km s^−1^ and *T* = 0.5 MK, with the fast solar wind flow ensuring a fast initialization. The IMF makes a 30° cone angle with the shock normal, similar to the subsolar shock in the global run, and the IMF strength is *B* = 5 nT in the run with Alfvén Mach number *M*_A_ = 6.9 or *B* = 8.6 nT in the run with *M*_A_ = 4. The shock is initialized at *x* = 0 in the de Hoffmann–Teller frame, and the downstream parameters are calculated using the Rankine–Hugoniot jump conditions, similarly to the shock simulations performed in ref. ^55^.

The top panels in Extended Data Figs. 6 and 7 show the density, magnetic field strength and magnetic field components in the simulation domain at *t* = 500 s from the beginning of these local runs. The shock position has moved towards negative *x* because the Rankine–Hugoniot jump conditions are based on magnetohydrodynamics and thus the shock is not in equilibrium when kinetic effects are at play. The upstream is filled with typical foreshock fast-mode waves that are carried towards the shock by the solar wind flow. The *B*_*x*_ component remains constant across the simulation domain to satisfy ∇⋅**B** = 0.

The dispersion plots in the bottom panels of Extended Data Figs. 6 and 7 are obtained by performing a 2D Fourier transform between *x* = −10*R*_E_ and *x* = −5*R*_E_ (black rectangles in panel b), and between *t* = 200 s and *t* = 500 s. As in the global simulation, a clear signal is observed in the magnetic field strength, consistent with downstream-oriented fast-mode waves (Extended Data Figs. 6d and 7d). These are accompanied by structures propagating with the bulk flow. Similar features are observed in the two local runs.

Regarding the transverse waves, appearing here on the *B*_*y*_ magnetic field component because the selected IMF is contained in the *x*−*z* plane, distinct signals for upstream- and downstream-oriented wavevectors are observed in the run with *M*_A_ = 4. This is consistent with the findings by Krauss-Varban et al.^28,29,52^, who identified the upstream-propagating waves as foreshock waves having been mode-converted to Alfvén waves through the shock. At higher Mach number, however, the distinction between the two oppositely propagating modes tends to disappear. This is consistent with the work by Quest^64^, who showed that the downstream wave velocity of the Alfvén waves becomes much smaller than the Alfvén speed as the Mach number increases, resulting in the waves’ being nearly non-convective. We also note that the mode-converted Alfvén waves are restricted to lower frequencies than the other modes, probably due to wave damping^29^, which makes it more difficult to identify them.

This suggests that foreshock waves interacting with the bow shock give rise to both mode-converted Alfvén waves and downstream-oriented fast-mode waves. The latter were not reported in the studies by Krauss-Varban et al.^28,29,52^, possibly because they focused on the transverse magnetic field component, on which these waves are not detectable (Extended Data Figs. 6e and 7e), whereas they are clearly seen in the total magnetic field strength (Extended Data Figs. 6d and 7d).

On the other hand, although the local 1D run clearly demonstrates the presence of upstream-oriented Alfvén waves in the downstream, these waves are challenging to observe in the global simulation. We do, however, observe a signal consistent with those waves when performing a Fourier transform of the transverse, *B*_*z*_, component (Fig. 3e, right), at low frequencies ($\omega \le 0.05{{{\varOmega }}}_{{{{\rm{ci}}}}}^{-1}$). This signal being much weaker in the global simulation than in the local runs could be due to the different properties of the downstream plasma: The subsolar magnetosheath flow is sub-Alfvénic in the global run, whereas the downstream remains super-Alfvénic in the local simulations. This could strongly affect the wave growth, as the sunwards-propagating Alfvén waves are effectively moving back towards the shock in the global simulation.

## Online content

Any methods, additional references, Nature Portfolio reporting summaries, source data, extended data, supplementary information, acknowledgements, peer review information; details of author contributions and competing interests; and statements of data and code availability are available at 10.1038/s41567-022-01837-z.

## Figures and Tables

**Extended Data Table 1:**
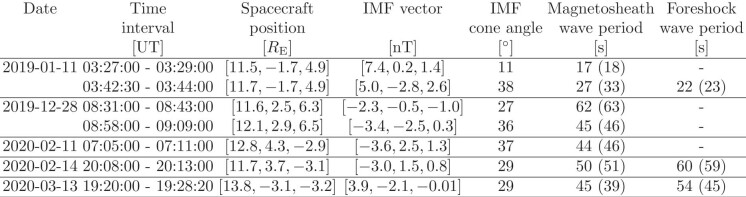
List of all events used in the spacecraft data analysis, including important parameters for each interval. Intervals during which the MMS satellites were located in the quasi-parallel subsolar magnetosheath. The upstream interplanetary magnetic field (IMF) vector and cone angle are obtained either from the OMNI data set or directly from measurements from the ACE or Wind spacecraft propagated to the bow shock, depending on the data availability for each event. The IMF cone angle provides a good estimate of the shock *θ*_Bn_ angle upstream of MMS, because the spacecraft are located in the subsolar region. The magnetosheath wave period is obtained as the peak of the power spectral density of the magnetic field strength during the interval that is closest to the predicted foreshock wave period, given between parentheses. The last column provides the observed (predicted) foreshock wave period when MMS probed the foreshock shortly before or after the magnetosheath interval. Those events for which no data were available in the foreshock are marked with ‘-’. Note that the solar wind and IMF conditions somewhat differ between the magnetosheath and foreshock intervals, hence the slightly different wave periods, but which show good agreement with the predicted values.

## Data Availability

The Vlasiator global run described here takes several terabytes of disk space and is kept in storage maintained within the CSC-IT Center for Science. It can be accessed through the following link: https://a3s.fi/swift/v1/AUTH_81f1cd490d494224880ea77e4f98490d/vlasiator-2d-afc. The data from the local 1D shock runs can be accessed through the following link: https://datacloud.helsinki.fi/index.php/s/NBFEj7TJf6oQjqN. Vlasiator uses a data structure developed in-house (https://github.com/fmihpc/vlsv/^65^), which can be read using the Analysator software https://github.com/fmihpc/analysator/^66,67^. Usage of Vlasiator data must comply with the data policy as described on the Vlasiator website (https://www.helsinki.fi/en/researchgroups/vlasiator/rules-of-the-road). The MMS data are publicly available and can be found on https://lasp.colorado.edu/mms/sdc/public/. Source data are provided with this paper.

## Source data

Source Data Fig. 2 Time series of the virtual satellite measurements extracted at different positions along the Sun–Earth line, used to generate Fig. 2. Each line corresponds to a given time stamp (first column) and a given virtual satellite position (second to fourth columns). Plotted in Fig. 2 are the total magnetic field strength (ninth column, B.magnitude), the proton density (13th column, proton/rho) and the magnetic field components (5th to 8th columns, B.x, B.y, B.z). The other panels of Fig. 2 are obtained from a wavelet transform of the magnetic field strength (c and h) and the wavelet cross-correlation of the magnetic field strength and proton density (d and i) and the wavelet transform of the magnetic field components parallel and perpendicular to the mean magnetic field (e and j).
Source Data Extended Data Fig. 1 Time series of the virtual satellite measurements extracted at *x* = 9.6*R*_E_, *y* = 0*R*_E_, used to generate Extended Data Fig. 1. The time stamps of the virtual satellite measurements are given in the first column. Plotted in Extended Data Fig. 1 are the total magnetic field strength calculated as the norm of the three components (5th to 8th columns, B.x, B.y, B.z), the proton density (9th column, proton/rho) and the magnetic field components (5th to 8th columns, B.x, B.y, B.z). The other panels of Extended Data Fig. 1 are obtained from a wavelet transform of the magnetic field strength (c) and the wavelet cross-correlation of the magnetic field strength and proton density (d)

## Code availability

Vlasiator http://www.helsinki.fi/en/researchgroups/vlasiator/^68,69^ is distributed under the GPL-2 open source license at http://github.com/fmihpc/vlasiator/^70^.
